# Effects of Dietary *Macleaya cordata* Extract Containing Isoquinoline Alkaloids Supplementation as an Alternative to Antibiotics in the Diets on Growth Performance and Liver Health of Broiler Chickens

**DOI:** 10.3389/fvets.2022.950174

**Published:** 2022-07-27

**Authors:** Yang Liu, Yang Li, Jiaxing Niu, Hua Liu, Ning Jiao, Libo Huang, Shuzhen Jiang, Lei Yan, Weiren Yang

**Affiliations:** ^1^Shandong Provincial Key Laboratory of Animal Biotechnology and Disease Control and Prevention, Department of Animal Science and Veterinary Medicine, Shandong Agricultural University, Taian, China; ^2^College of Animal Science and Technology, Hunan Agriculture University, Changsha, China; ^3^Shandong New Hope Liuhe Group Co., Ltd., Qingdao, China

**Keywords:** antimicrobial growth promoter, broiler, growth performance, liver health, plant extract

## Abstract

This study aimed to investigate the effects of dietary supplementation with *Macleaya cordata* extract (MCE) containing protopine and allotypotopine on the growth performance and liver health in broiler chickens. A total of 486 1-day-old male AA broiler chickens were randomly assigned to the following three groups: (1) control (CON) group, broiler chickens fed a basal diet; (2) AGP group (positive control), broiler chickens fed a basal diet supplemented with 50 mg/kg aureomycin; (3) MCE group, broiler chickens fed a basal diet supplemented with 0.6 mg/kg MCE including 0.4 mg/kg protopine and 0.2 mg/kg allotypotopine. The results showed that the MCE group had significantly higher final body weight and average daily gain from d 0 to 42 than the other groups (*p* < 0.05), and groups MCE and AGP both had significantly lower feed-to-gain ratio from d 0 to 42 than the CON group (*p* < 0.05). Serum total protein, high-density lipoprotein cholesterol, low-density lipoprotein cholesterol, total cholesterol, glucose, immunoglobulin A, immunoglobulin M, and complements (C3, C4) concentrations in the MCE group were significantly higher than in the CON group (*p* < 0.05). Dietary MCE or aureomycin supplementation significantly reduced the hepatic contents of 8-hydroxy-2'-deoxyguanosine, malondialdehyde, interleukin (IL)-1β, IL-6, NLRs family pyrin domain containing 3 (NLRP3), and caspase-1 in the liver (*p* < 0.05). Moreover, MCE or aureomycin supplementation significantly inhibited mRNA expressions of Toll-like receptor 4, myeloid differentiation factor 88, nuclear factor-κB, and NLRP3, as well as the expression ratio of Bax to Bcl-2 mRNA (*p* < 0.05). Therefore, our study suggested that dietary supplementation with 0.6 mg/kg MCE containing protopine and allocryptopine improved growth performance and benefited liver health in broiler chickens possibly through inhibiting caspase-1-induced pyroptosis by inactivating TLR4/MyD88/NF-κB/NLRP3 signaling pathway, and provided support for the application of MCE containing protopine and allocryptopine as an alternative to antibiotics in the feed industry.

## Introduction

With the continuous expansion of the scale of intensive farming, the disease factors that endanger poultry production are becoming increasingly serious, which raises the risk of injury to broiler chickens (1–3). The liver is the largest substantive gland and an important metabolic organ, playing vital roles in excretion, metabolism, detoxification, and production of various coagulation factors. Liver injury in poultry is a common clinical disease, which could induce decreased poultry performance and even death, resulting in a huge economic loss (1). Antibiotic treatment is an effective way to protect the liver (4). Currently, due to the prohibition of antimicrobial growth promoter (AGP), looking for alternatives to antibiotics is of great significance to guarantee liver function and animal production (5, 6).

A large number of studies have proved that plant extract is a good substitute for AGP (7). *Macleaya cordata* belongs to the *Papaveraceae* family and is mainly distributed in central and southeastern China (8). *Macleaya cordata* extract (MCE) has been reported to have a broad spectrum of biological activities, such as anti-viral, anti-inflammatory, antimicrobial, and detoxifying effects (9). This is mainly attributed to its active principles including benzophenanthridine alkaloids (sanguinarine and chelerythrine) and isoquinoline alkaloids (protopine and allocryptopine) (10). Sanguinarine and chelerythrine from MCE have been exploited as natural feed additives in the European Union, and are widely used as substitutes for AGP in poultry and livestock production (11, 12). A previous study also demonstrated that protopine and allocryptopine have the beneficial biological activities of anti-inflammatory, anti-bacterial, and hepatoprotective effects (13). However, there is little literature evaluating the effects of dietary supplementation with MCE containing protopine and allocryptopine on the growth performance and health status of broiler chickens.

Therefore, this study was conducted to explore the effects of dietary supplementation with MCE containing protopine and allocryptopine on the growth performance and liver health in broiler chickens and to highlight the application of MCE containing isoquinoline alkaloids as an effective alternative to AGP.

## Materials and Methods

### Experimental Design and Management

A total of 486 newly hatched male Arbor Acres broiler chickens with average body weight (BW) of 48.76 ± 0.25 g were obtained from a local hatchery and randomly assigned to three dietary treatments according to their BW with 6 replicates per treatment in a 42-d study. Each replicate contained 27 chickens housed in a three-level cage. The three treatment groups were designed as follows: (1) control (CON) group, broiler chickens fed a basal diet; (2) AGP group (positive control), broiler chickens fed a basal diet supplemented with 50 mg/kg aureomycin (14); (3) MCE group, broiler chickens fed a basal diet supplemented with 0.6 mg/kg MCE including 0.4 mg/kg protopine and 0.2 mg/kg allotypotopine. The MCE was provided by the Micolta Bioresource Company Limited (Changsha 410331, PR China) and premixed with starch before supplementation. The 42-day trial spanned two phases including the starter period (days 0–21) and the grower period (days 21–42). The basal diets (Table 1) were formulated to respect the nutritional requirements of broiler chickens recommended by the Ministry of Agriculture of P.R. China (2004). Feed and water were offered *ad libitum* during the experiment. All broiler chickens were housed in a temperature- and light-controlled room with continuous light at the Shandong Agricultural University. The room temperature was kept at 32–34°C for the first week and then reduced by 3°C per week until it reached 21°C. In addition, all broiler chickens were vaccinated with inactivated Newcastle disease vaccine at 7 days of age and inactivated infectious bursal disease vaccine at 14 days of age. Feed intake per replicate was recorded daily to calculate average daily feed intake (ADFI), and all broilers were weighed by replicates at 21 and 42 days to calculate average daily gain (ADG). Then the feed-to-gain ratio (F/G) was obtained based on feed intake and BW gain.

**Table 1:** Ingredients composition and nutrient levels of basal diets (as-fed basis).

**Items**	**Phases**	
	**0–21 d**	**21–42 d**
**Ingredients, %**		
Corn	55.91	55.91
Soybean meal, 44% CP	13.78	10.18
Wheat bran	11.98	12.98
Corn starch residue	7.99	9.98
Corn gluten meal	3.99	3.99
Extruded soybean	1.50	2.10
Limestone	1.70	1.70
Calcium monophosphate	1.10	1.10
L-Lysine HCl, 76.8%	1.00	1.00
DL-Methionine, 98%	0.20	0.20
L-Threonine, 98%	0.10	0.10
Sodium chloride	0.40	0.40
Choline	0.10	0.10
Phytase	0.10	0.10
Complex enzyme	0.02	0.02
Trace mineral premix^a^	0.10	0.10
Vitamin premix^b^	0.02	0.02
Antioxidant	0.02	0.02
Total	100	100
**Calculated analysis, %**		
Metabolizable energy,MJ/kg	12.33	12.50
Crude protein	19.47	17.93
Crude fat	3.45	3.74
Calcium	0.94	0.87
Available phosphorus	0.35	0.33
Lysine	1.15	1.00
Methionine	0.50	0.40

a *Provided per kilogram of complete basal diet: 10 mg of Cu as CuSO_4_, 100 mg of Fe as FeSO_4_, 1.1 mg of I as Ca(IO_3_)_2_, 65 mg of Zn as ZnSO_4_, 100 mg of Mn as MnSO_4_ and 0.3 mg of Se as Na_2_SeO_3_*.

b *Provided per kilogram of complete basal diet: vitamin A 10,000 IU, vitamin D_3_ 3,000 IU, vitamin E 30 IU, menadione 1.3 mg, thiamine 2.2 mg, riboflavin 8 mg, pyridoxine 4 mg, vitamin B_12_ 0.025 mg, D-biotin 0.2 mg, niacin 40 mg, folic acid 1 mg and D-calcium pantothenate 10 mg*.

### Samples Collection

On day 42, one broiler chicken per replicate of body weight close to the average BW of the replicates was selected for sampling after a 12-h fast. A blood sample (5 mL) was collected from the wing vein using a coagulation-promoting vacutainer tube and kept in a slanting position for 30 min. The serum was obtained after centrifugation at 3,000 *g* for 15 min at 4°C and stored at −20°C. Subsequently, the selected broiler chickens were euthanized and then dissected under aseptic conditions. The liver of each chicken was quickly removed, frozen in liquid nitrogen, and stored at −80°C until further analysis.

### Analysis of Biochemical Parameters Levels in Serum

The serum concentrations of the total protein (TP), albumin (ALB), high-density lipoprotein cholesterol (HDL), low-density lipoprotein cholesterol (LDL), urea nitrogen (UREA), total cholesterol (TCHO), triglyceride (TG), and glucose (GLU) were determined using commercial kits (Nanjing Jiancheng Bioengineering Institute, Nanjing, China) on an automatic clinical chemistry analyzer (Roche, Cobus-MiraPlus, Roche Diagnostic System Inc., United States).

### Analysis of Immunoglobulins and Complements Concentrations in Serum

The concentrations of immunoglobulin A (IgA), immunoglobulin G (IgG), immunoglobulin M (IgM), complement C3, and complement C4 in the serum were determined by using commercial ELISA kits (Jiangsu Meimian Industrial Co., Ltd, Jiangsu, China) according to the procedure for ELISA described in Chen et al. (15).

### Analysis of Biomarkers of Liver Health

Serum alanine transaminase (ALT) activity and hepatic 8-hydroxy-2'-deoxyguanosine (8-OHdG) and malondialdehyde (MDA) concentrations were detected using the commercial kits (Nanjing Jiancheng Bioengineering Institute). The determination of ALT was performed on the Cobus-MiraPlus chemistry analyzer (Roche). The concentration of 8-OHdG in the liver was determined according to the manufacturer's protocol as described in the previous study (16). The MDA concentration in the liver was measured using a specific thiobarbituric acid method with the kit (Nanjing Jiancheng Bioengineering Institute) as previously described (17).

### Analysis of Hepatic Inflammatory Factors and Caspases Levels

The levels of tumor necrosis factor-α (TNF-α), interleukin-1β (IL-1β), interleukin-6 (IL-6), interleukin-18 (IL-18), NLRs family pyrin domain containing 3 (NLRP3), and caspase-1 and caspase-3 in the liver samples were determined using ELISA kits (Jiangsu Meimian Industrial Co., Ltd) following the protocol described in a previous study (15).

### Analysis of Gene Expression

The total RNA of liver samples was extracted using the AG RNAex Pro reagent (Accurate Biology, Hunan, China). The cDNA was acquired by the reverse transcription (RT) kit (Accurate Biology, Hunan, China). The cDNA samples were amplified by real-time quantitative polymerase chain reaction with SYBR^®^ Green Premix Pro Taq HS qPCR Kit (AG11701, Accurate Biology, Dalian, China). The mRNA expression levels of Toll-like receptor 4 (*TLR4*), myeloid differentiation factor 88 (*MyD88*), nuclear factor-κB (*NF-*κ*B*), *NLRP3*, sirtuin1 (*Sirt1*), nuclear factor erythroid 2-related factor 2 (*Nrf2*), heme-oxygenase 1 (*HO-1*), superoxide dismutase 1 (*SOD1*), superoxide dismutase 2 (*SOD2*), catalase (*CAT*), glutathione peroxidase 1 (*GPX1*), NAD(P)H quinone oxidoreductase-1 (*NQO1*), B-cell lymphoma-2 (*Bcl-2*), and Bcl-2-associated X (*Bax*) in the liver samples were assessed using a LightCycler 96 (Roch, Switzerland) with SYBR^®^ Green Premix Pro Taq HS qPCR Kit (AG11701, Accurate Biology, Da Lian, China). All the primer sequences are listed in Table 2. β*-actin* was used as an internal reference gene, and the 2^−ΔΔCT^ method was used to calculate the messenger ribonucleic acid (mRNA) expression of target genes relative to β*-actin* (17).

**Table 2:** Primer sequences used for quantitative real-time PCR.

**Genes^b^**	**GenBank**	**Primer sequences, 5′-3′^a^**	**Size, bp**
*β-actin*	NM_205518.1	F:ATTGTCCACCGCAAATGCTTC R:AAATAAAGCCATGCCAATCTCGTC	113
*TLR4*	NM_001030693.2	F:CATCTCTGGAGTTCCTGCTGAA R:TGTATGGATGTGGCACCTTGA	145
*MyD88*	NM_001030962.5	F:CGGAGGATGGTGGTCGTCATT R:TCGTTCTTCATGGTCTTGCACTTG	140
*NF-κB*	NM_001396038.1	F:CAGCCCATCTATGACAACCG R:TCAGCCCAGAAACGAACCTC	152
*NLRP3*	NM_001348947.2	F:GAAGGTGCTGCTATGGACATTG R:CGTGCTCTGTGTATTTCTGCTTAT	118
*Sirt1*	XM_046920057.1	F:GATCAGCAAAAGGCTGGATGGT R:ACGAGCCGCTTTCGCTACTAC	143
*Nrf2*	XM_015289381.2	F:CCCGCACCATGGAGATCGAG R:GGAGCTGCTCTTGTCTTTCCT	180
*HO-1*	NM_205344.1	F:GTCGTTGGCAAGAAGCATCC R:GGGCCTTTTGGGCGATTTTC	106
*SOD1*	NM_205064.2	F:CGCAGGTGCTCACTTCAATCC R:CAGTCACATTGCCGAGGTCAC	89
*SOD2*	NM_204211.2	F:GCTGTATCAGTTGGTGTTCAAGGA R:GCAATGGAATGAGACCTGTTGTTC	130
*CAT*	NM_001031215.2	F:GGAGGTAGAACAGATGGCGTATG R:CGATGTCTATGCGTGTCAGGAT	114
*GPX1*	NM_001277853.3	F:CGGCTTCAAACCCAACTTCAC R:CTCTCTCAGGAAGGCGAACAG	85
*NQO1*	NM_001277619.2	F:GAGTGCTTTGTCTACGAGATGGA R:ATCAGGTCAGCCGCTTCAATC	104
*Bax*	XM_422067	F:TGAGCATGTAGCAACGGAAG R:AGCAAGCTGATTGACGGTCT	295
*Bcl-2*	NM_205339.3	F:AGGACAACGGAGGATGGGATG R:CACCAGAACCAGGCTCAGGAT	110

a *F: forward primer; R: reverse primer*.

b *TLR4, Toll-like receptor 4; MyD88, myeloid differentiation primary response 88; NF-κB, nuclear factor-kappa B; NLRP3, Nod-like receptor protein 3; Sirt1, sirtuin1; Nrf2, nuclear factor erythroid 2-related factor 2; HO-1, heme-oxygenase 1; SOD1, superoxide dismutase 1; SOD2, superoxide dismutase 2; CAT, catalase; GPX1, glutathione peroxidase 1; NQO1, NAD(P)H quinone oxidoreductase 1; Bax, Bcl-2-associated X; Bcl-2, B-cell lymphoma-2*.

### Statistical Analysis

The data were statistically analyzed using a one-way analysis of variance (ANOVA) of SAS 9.4 (Institute Inc., Cary, NC, United States) after the normality of data was assessed using Shapiro-Wilk's statistic (W > 0.05). Multiple comparisons of treatment means were conducted using the least significant difference test. The results in the figures are expressed as the mean ± standard error. Significant differences are represented in the figures by ^*^p < 0.05, ^**^p < 0.01, and ^***^p < 0.001, and ^#^0.05 ≤*p* < 0.1 is considered as a trend toward significance.

## Results

### Effect of MCE on Growth Performance

**Figure 1:**
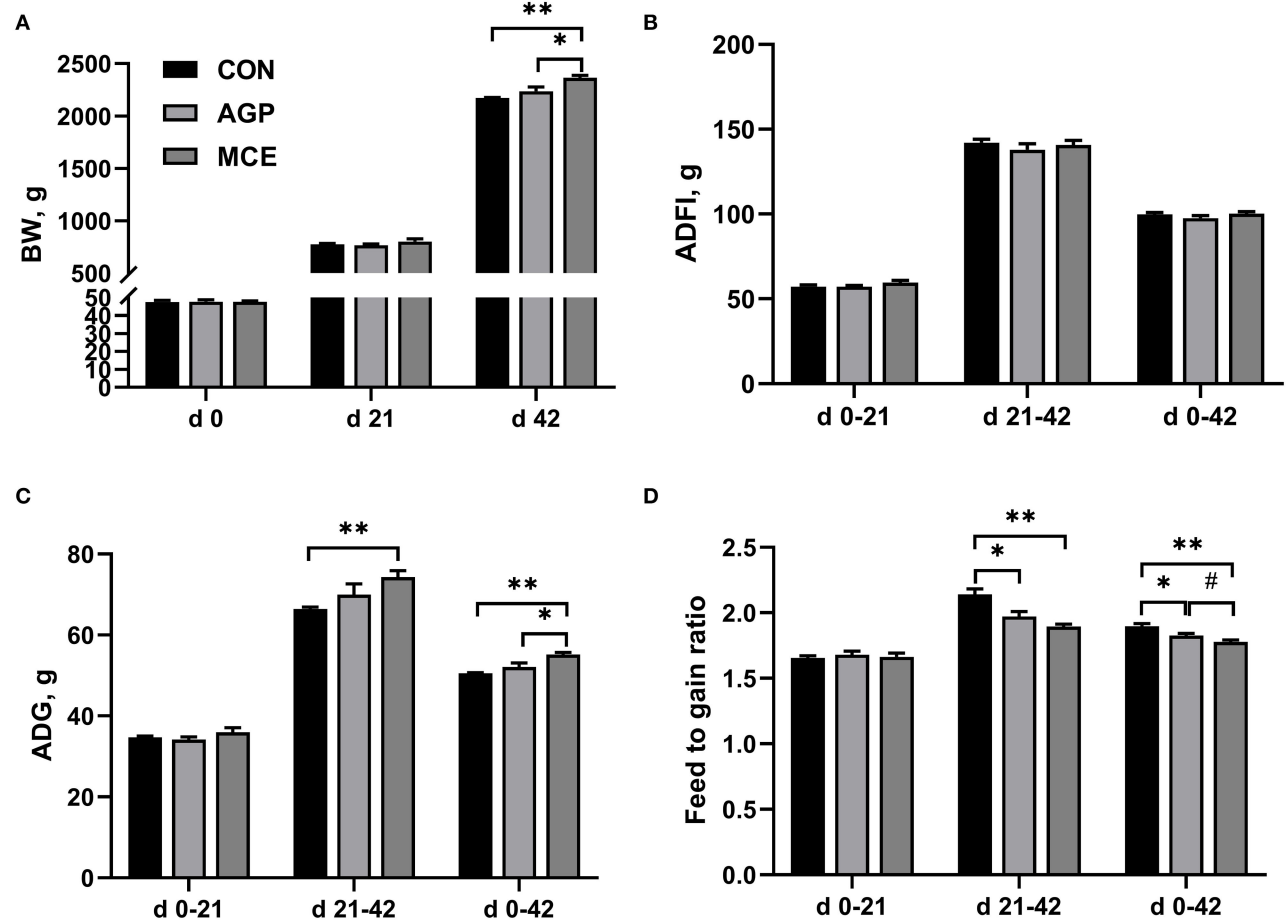
Effects of dietary supplemented with MCE on growth performance of broiler chickens. **(A)** Body weight (BW); **(B)** Average daily feed intake (ADFI); **(C)** Average daily gain (ADG); **(D)** Feed to gain ratio. CON, broiler chickens fed basal diet; AGP group, broiler chickens fed a basal diet supplemented with 50 mg/kg aureomycin; MCE, broiler chickens fed basal diet supplemented with 0.6 mg/kg *Macleaya cordata* extract (MCE) containing protopine and allocryptopine. Values are mean ± standard error (*n* = 6). ^#^0.05 ≤ *p* < 0.10, *p < 0.05, **p < 0.01.

The effects of MCE supplementation on the growth performance of broiler chickens are shown in Figure 1. The broilers in the MCE group had the highest BW on day 42 of the experiment, which was significantly higher than the broilers in the CON group and AGP group (*p* < 0.05). No significant differences were observed in ADFI throughout the experiment among the three groups (*p* > 0.05). From d 21 to 42, ADG in the MCE group was significantly higher than that in the CON group (*p* < 0.05); from d 0 to 42, significantly higher ADG in the MCE group was observed compared with the CON group and AGP group (*p* < 0.05). Besides, from d 21 to 42, broilers in the AGP group and MCE group had significantly lower F/G than the broilers in the CON group (*p* < 0.05); from d 0 to 42, F/G in the AGP group and MCE group was significantly lower than that in CON group (*p* < 0.05), and F/G in MCE group tended to be lower than that in AGP group (*p* < 0.10).

### Effect of MCE on Serum Biochemical Parameters

**Figure 2:**
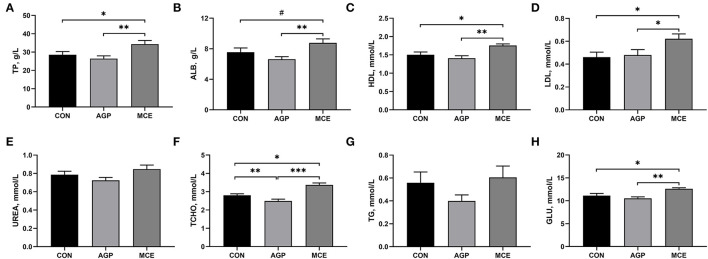
Effects of dietary supplemented withMCE on serum biochemical parameters of broiler chickens. **(A)** Total protein (TP); **(B)** Albumin (ALB); **(C)** High density lipoprotein (HDL); **(D)** Low-density lipoprotein (LDL); **(E)** Urea nitrogen (UREA); **(F)** Total-cholesterol (TCHO); **(G)** Triglyceride (TG); **(H)** Glucose (GLU). CON, broiler chickens fed basal diet; AGP group, broiler chickens fed a basal diet supplemented with 50 mg/kg aureomycin; MCE, broiler chickens fed basal diet supplemented with 0.6 mg/kg *Macleaya cordata* extract (MCE) containing protopine and allocryptopine. Values are mean ± standard error (*n* = 6). ^#^0.05 ≤ *p* < 0.10, *p < 0.05, **p < 0.01, ***p < 0.001.

As shown in Figure 2, the serum concentrations of TP, HDL, LDL, and GLU were significantly higher in the MCE group than in the CON group and AGP group (*p* < 0.05). The MCE group had significantly higher ALB than the AGP group (*p* < 0.05), and tended to have higher ALB concentration than the CON group (*p* < 0.10). Broilers in the MCE group had significantly higher TCHO concentration than broilers in the CON group (*p* < 0.05), and the TCHO concentration in the AGP group was significantly lower than that in the CON group (*p* < 0.05). Besides, no significant differences were observed in the UREA and TG concentrations among the three treatments (*p* > 0.05).

### Effect of MCE on Serum Immunoglobulins and Complements Concentrations

**Figure 3:**
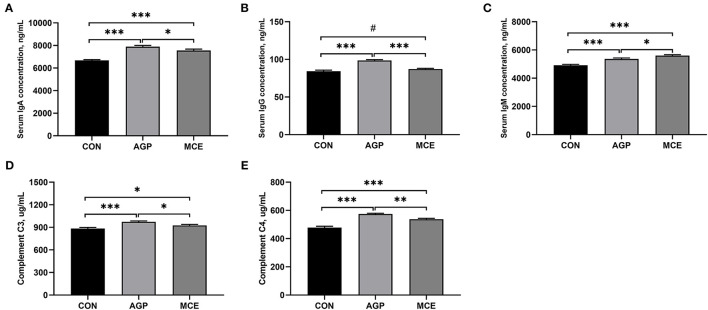
Effects of dietary supplemented with MCE on serum immunoglobulins and complements concentrations of broiler chickens. **(A)** Immunoglobulin A (IgA); **(B)** Immunoglobulin G (IgG); **(C)** Immunoglobulin M (IgM); **(D)** Complement C3; **(E)** Complement C4. CON, broiler chickens fed basal diet; AGP group, broiler chickens fed a basal diet supplemented with 50 mg/kg aureomycin; MCE, broiler chickens fed basal diet supplemented with 0.6 mg/kg *Macleaya cordata* extract (MCE) containing protopine and allocryptopine. Values are mean ± standard error (*n* = 6). ^#^0.05 ≤ *p* < 0.10, *p < 0.05, **p < 0.01, ***p < 0.001.

As shown in Figure 3, the serum concentrations of IgA, IgM, C3, and C4 were significantly higher in the MCE group and AGP group than in the CON group (*p* < 0.05). The MCE group had significantly lower IgA, C3, and C4 concentrations (*p* < 0.05), but had significantly higher IgM concentration than the AGP group (*p* < 0.05). Besides, the AGP group had significantly higher IgG than the CON group and MCE group (*p* < 0.05), and IgG concentration in the MCE group tended to be higher than that in the CON group (*p* < 0.10).

### Effect of MCE on Biomarkers of Liver Health

**Figure 4:**
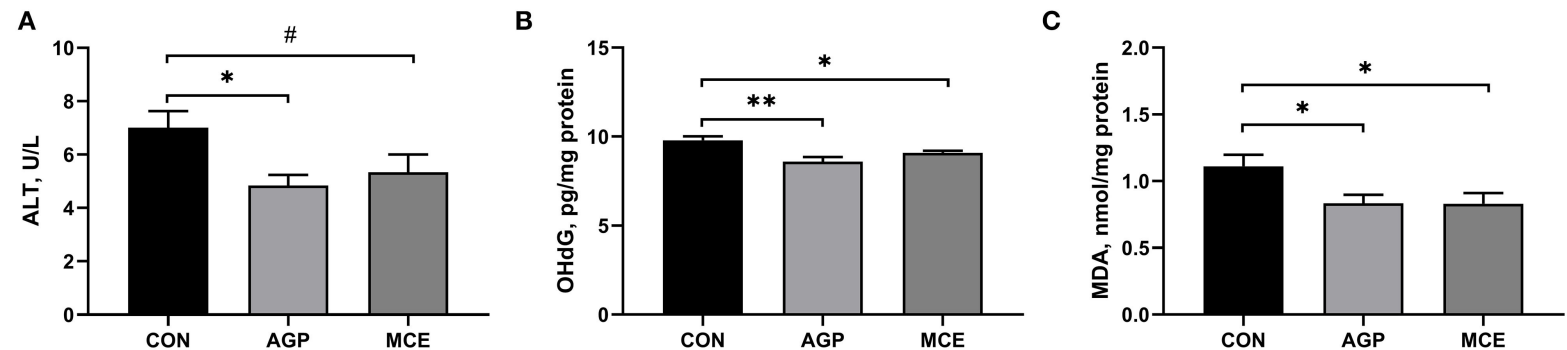
Effects of dietary supplemented with MCE containing protopine and allocryptopine on biomarkers of liver health of broiler chickens. **(A)** Alanine transaminase (ALT); **(B)** 8-hydroxy-2'-deoxyguanosine (8-OHdG); **(C)** Malondialdehyde (MDA). CON, broiler chickens fed basal diet; AGP group, broiler chickens fed a basal diet supplemented with 50 mg/kg aureomycin; MCE, broiler chickens fed basal diet supplemented with 0.6 mg/kg *Macleaya cordata* extract (MCE) containing protopine and allocryptopine. Values are mean ± standard error (*n* = 6). ^#^0.05 ≤ *p* < 0.10, *p < 0.05, **p < 0.01.

The effects of MCE on hepatic health biomarkers are shown in Figure 4. Broilers in the AGP group had significantly lower serum ALT concentration than broilers in the CON group (*p* < 0.05), and serum ALT concentration in the MCE group tended to be lower than that in the CON group (*p* < 0.10). Besides, the liver concentrations of 8-OHdG and MDA were significantly lower in the MCE group and AGP group than in the CON group (*p* < 0.05).

### Effects of MCE on Hepatic Inflammatory Factors and Caspases Levels

**Figure 5:**
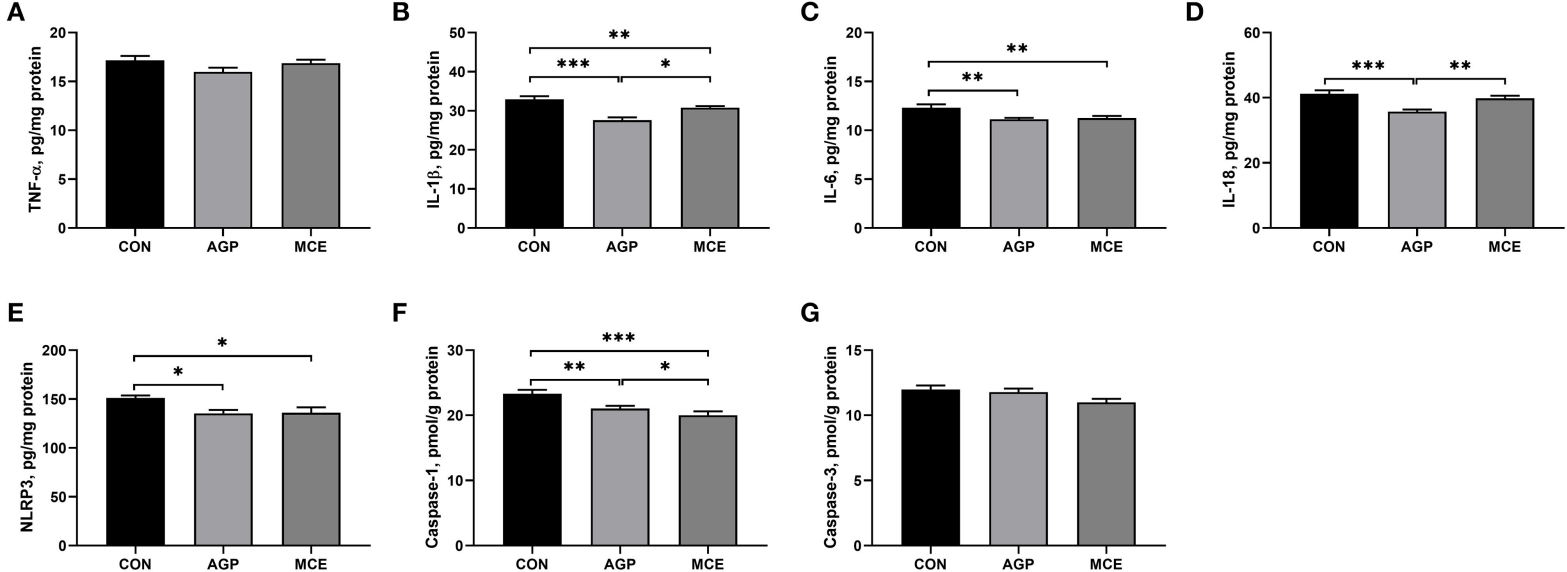
Effects of dietary supplemented with MCE on hepatic inflammatory factors and caspases levels of broiler chickens. **(A)** Tumor necrosis factor-α (TNF-α); **(B)** Interleukin-1β (IL-1β); **(C)** Interleukin-6 (IL-6); **(D)** Interleukin-18 (IL-18); **(E)** NLRs family pyrin domain containing 3 (NLRP3); **(F)** Caspase-1; **(G)** Caspase-3. CON, broiler chickens fed basal diet; AGP group, broiler chickens fed a basal diet supplemented with 50 mg/kg aureomycin; MCE, broiler chickens fed basal diet supplemented with 0.6 mg/kg *Macleaya cordata* extract (MCE) containing protopine and allocryptopine. Values are mean ± standard error (*n* = 6). *p < 0.05, **p < 0.01, ***p < 0.001.

The effects of MCE on hepatic inflammatory factors and caspases levels are shown in Figure 5. The liver concentration of IL-1β was significantly lower in the MCE group and AGP group than in the CON group (*p* < 0.05), and the MCE group showed significantly higher hepatic IL-1β concentration than the AGP group (*p* < 0.05). The MCE group and AGP group had significantly lower IL-6 and NLRP3 concentrations than the CON group (*p* < 0.05), and the AGP group had significantly lower IL-18 concentration than the CON group and MCE group in the liver (*p* < 0.05). The liver concentration of caspase-1 was significantly lower in the MCE group and AGP group than in the CON group (*p* < 0.05), and the MCE group showed significantly higher serum concentration than the AGP group (*p* < 0.05). No significant differences were observed in the liver TNF-α and caspase-3 concentrations among the three groups (*p* > 0.05).

### Effects of MCE on Genes Expressions in Liver

**Figure 6:**
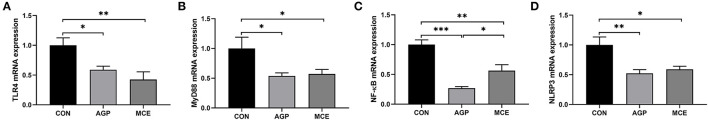
Effects of dietary supplemented with MCE on expressions of inflammatory genes in liver of broiler chickens. **(A)** Toll-like Receptor 4 (TLR4); **(B)** Myeloid differentiation primary response 88 (MyD88); **(C)** Nuclear factor-kappa B (NF-κB); **(D)** NLRs family pyrin domain containing 3 (NLRP3). CON, broiler chickens fed basal diet; AGP group, broiler chickens fed a basal diet supplemented with 50 mg/kg aureomycin; MCE, broiler chickens fed basal diet supplemented with 0.6 mg/kg *Macleaya cordata* extract (MCE) containing protopine and allocryptopine. Values are mean ± standard error (*n* = 6). *p < 0.05, **p < 0.01, ***p < 0.001.

The expressions of inflammatory genes are shown in Figure 6. The mRNA expressions of *TLR4, MyD88, NF-*κ*B*, and *NLRP3* were significantly lower in the MCE group and AGP group than in the CON group (*p* < 0.05), and the MCE group showed significantly higher *NF-*κ*B* mRNA expression than the AGP group (*p* < 0.05).

**Figure 7:**
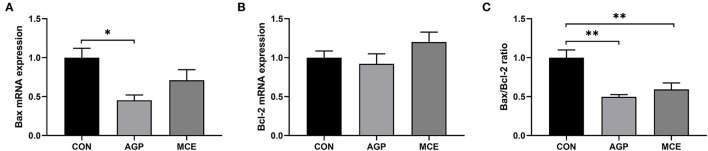
Effects of dietary supplemented with MCE on apoptosis genes in livers of broiler chickens. **(A)** BCL2-Associated X (Bax); **(B)** B-cell lymphoma-2 (Bcl-2); **(C)** Bax/Bcl-2 ratio. CON, broiler chicken fed basal diet; AGP group, broiler chickens fed a basal diet supplemented with 50 mg/kg aureomycin; MCE, broiler chickens fed basal diet supplemented with 0.6 mg/kg *Macleaya cordata* extract (MCE) containing protopine and allocryptopine. Values are mean ± standard error (*n* = 6). *p < 0.05, **p < 0.01.

The gene expressions of apoptosis regulators are shown in Figure 7. The mRNA expression of *Bax* was significantly lower in the AGP group than in the CON group (*p* < 0.05), and groups MCE and AGP showed significantly lower mRNA *Bax*/*Bcl-2* ratio than the CON group (*p* < 0.05). No significant difference was observed in the *Bcl-2* mRNA expression (*p* > 0.05).

**Figure 8:**
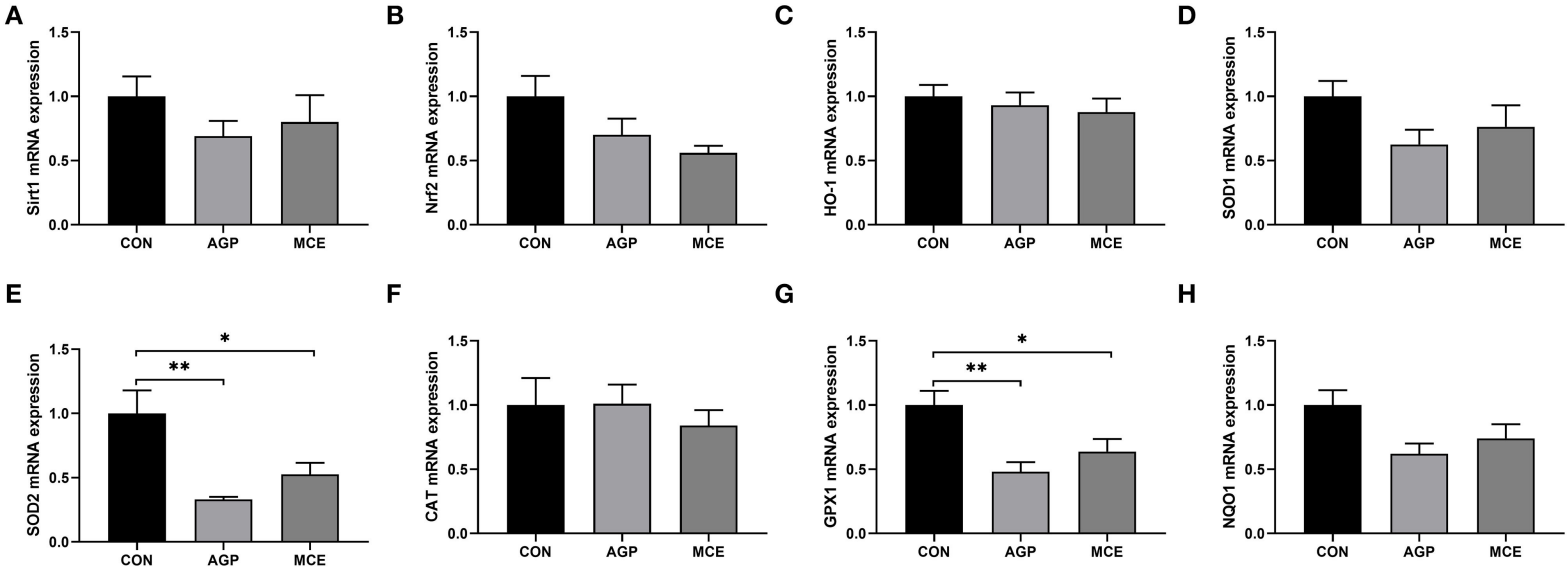
Effects of dietary MCE supplementation on expressions of antioxidation-related genes in livers of broiler chickens. **(A)** Sirtuin1 (Sirt1); **(B)** Nuclear factor erythroid 2-related factor 2 (Nrf2); **(C)** Heme-oxygenase 1 (HO-1); **(D)** Superoxide dismutase 1 SOD1; **(E)** Superoxide dismutase 2 (SOD2); **(F)** Catalase (CAT); **(G)** Glutathione peroxidase 1 (GPX1); **(H)** NAD(P)H: quinone oxidoreductase-1 (NQO1). CON, broiler chickens fed basal diet; AGP group, broiler chickens fed a basal diet supplemented with 50 mg/kg aureomycin; MCE, broiler chickens fed basal diet supplemented with 0.6 mg/kg *Macleaya cordata* extract (MCE) containing protopine and allocryptopine. Values are mean ± standard error (*n* = 6). *p < 0.05, **p < 0.01.

The expressions of antioxidation-related genes are shown in Figure 8. The mRNA expressions of *SOD2* and *GPX1* were significantly lower in the AGP group and MCE group than in the CON group (*p* < 0.05), and no significant differences were observed in the mRNA expressions of *Sirt1, Nrf2, HO-1, SOD1, CAT*, and *NQO1* among the groups (*p* > 0.05).

## Discussion

In this study, we found that broilers fed the MCE diet showed higher BW and ADG and lower feed-to-gain ratio than those fed the CON diet and AGP diet. A previous study showed that supplementation of *Chelidonium majus* extracts containing isoquinoline, caffeic acid derivatives, and phenolic acid in the diet improved BW and feed conversion ratio (FCR) and enhanced the digestibility of nutrients of broilers (18). A study in post-weaned piglets also demonstrated that supplemented with 120 mg/kg MCE provided at 12.5 g/kg mixture of quaternary-benzo(c)phenanthridine alkaloids and isoquinoline alkaloids in the diet increased BW gain, FCR, and nutrient digestibility, but did not affect jejunal morphology (19). Besides, we also found that aureomycin as AGP supplemented in the broiler diet could increase BW and ADG, and decrease the feed-to-gain ratio, which was in accord with previous studies (20, 21). Therefore, our study suggested that MCE-containing isoquinoline alkaloids supplementation could improve the growth performance of broiler chickens, and MCE-containing isoquinoline alkaloids had the potential to replace AGP in broiler diets.

The liver health and function play an important role in guaranteeing a good growth performance of broilers (22). A study has indicated that AGP (such as aureomycin) has the function of protecting the liver in poultry production (23). We found that MCE supplementation decreased serum ALT activity and hepatic 8-OhdG concentration in this study. Alanine transaminase is an important transaminase in hepatocytes. When the liver is damaged, ALT will be released into the bloodstream (24). An increase in serum ALT often suggested cellular (hepatocyte) damage (25). 8-Hydroxy-2'-deoxyguanosine is considered the most important indicator of DNA damage, as DNA damage can produce 8-OHdG in the nucleotide pool during DNA replication (26). A previous study has reported that higher hepatic 8-OHdG concentration is observed in aflatoxin B_1_-damaged liver in broiler chickens (27). Moreover, in this study, MCE supplementation in the diet significantly increased the serum contents of TP, ALB, GLU, HDL, LDL, and TCHO in broiler chickens. Liver is a dynamic organ in serum protein synthesis, gluconeogenesis, lipogenesis, and cholesterol metabolism (28). Serum TP and ALB are regarded as the important indicators of the protein metabolism, and decreased serum TP and ALB concentrations is usually related to malnutrition and growth retardation in animals (29, 30). The serum level of GLU is an indicator of glycolipid metabolism. The high serum GLU content observed in broilers in the MCE group might benefit to promote the digestion and absorption of carbohydrates, which was consistent with the results by Guo et al. (31). Serum levels of HDL, LDL, and TCHO are three important indicators of cholesterol metabolism. Generally, LDL is in charge of carrying TCHO and TG from the liver to peripheral tissues, and concerned with the risk of atherosclerosis and hyperuricemia; HDL is responsible for transporting TCHO from extrahepatic tissue to liver and plays an important role in alleviating atherosclerosis and the processes of antioxidant, anti-inflammatory, and anti-apoptotic properties (32). In this study, the addition of MCE to the feed significantly elevated the serum levels of IgA and IgM. Wang et al. (33) also reported that MCE supplementation could increase serum concentrations of IgA and IgM. Serum IgA and IgM are important parts of humoral immunity (34). Besides, our results also showed increased serum complements C3 and C4 concentrations in the broilers fed the diet supplemented with MCE. Complement is a glycoprotein with enzymatic activity, which participates in the immune regulation of the body (35). A previous study also demonstrated that immunoglobulin could stimulate the increase in complement components, thereby enhancing the immune mechanism of the liver and protecting the liver from infection (36). C3 deficiency was often accompanied by diminished liver regeneration (37). Thorgersen et al. also found that inflammatory injury to the liver can cause a decrease in the levels of C3 and C4 (35). Based on the above, the higher serum levels of TP, ALB, HDL, LDL, TCHO, and GLU in the MCE group in this study might be attributed to the more intense metabolism during the growth process and development of broiler chickens, which helped to promote the growth performance (38), and MCE as an alternative to antibiotics supplementation in the diet might benefit to liver health of broilers in this study.

To further explore the mechanism underlying the protective effect of MCE on the liver, inflammatory factor concentrations and TLR4/MyD88/NF-κB/NLRP3 signaling pathway were evaluated. Accumulating evidence has proven that inflammatory response is an important factor triggering liver damage (39). In this study, the addition of MCE or aureomycin to the diet was found to reduce the levels of IL-1β and IL-6 in the liver of broilers. Song et al. (40) found that supplementation of aureomycin reduced serum concentrations of TNF-α, IL-2, and IL-6 in broiler chickens. The immune response is controlled by the complex interaction of various cytokines, and pro-inflammatory factors IL-6 and IL-1β have been used as markers of the inflammatory response (29). IL-1β plays a decisive role in the production of pro-inflammatory and homeostatic functions (41), while IL-6 is a multifunctional cytokine that can participate in the regulation of body inflammation, hematopoiesis, immune response, and acute phase response (42, 43). Previous studies have demonstrated that dietary supplementation of MCE reduces serum concentrations of IL-6 and IL-1β and inhibits the progress of the inflammatory disease (44). These results indicated that the hepatoprotective effect of MCE is partially attributed to the reduction of pro-inflammatory stimulus. The TLR4/MyD88/NF-κB/NLRP3 signaling pathway plays an important role in the activation of inflammation (45). Toll-like receptor 4, a member of toll-like receptor family, belongs to the family of pattern recognition receptor that plays a key role in the activation of innate immune response participating in response to a wide range of pathogen infection (46). Studies have shown that TLR4 can activate the NF-κB signaling pathway *via* the MyD88 protein to induce inflammatory response (47), rapidly inducing the activation of NLRP3 and the generation of a variety of cytokines involved in immune and inflammatory responses (48). A previous study showed that it helped liver protection with the inhibition of TLR4/MyD88/NF-κB signaling pathway (49). Lin et al. showed that the active ingredients in MCE could inhibit the activation of NF-κB and phosphorylation of the NF-κB subunit (IκBα) (9). In this study, dietary MCE or aureomycin supplementation decreased the mRNA levels of *TLR4, MyD88*, and *NF-*κ*B* in the livers of broiler chickens, suggesting that MCE and aureomycin could partially inhibit hepatic inflammatory by suppressing the TLR4/MyD88/NF-κB signaling pathway. A previous study in piglets also showed that olaquindox and aureomycin supplementation decreased the toll-like receptor expression in the ileum (50). Besides, in this study, dietary MCE supplementation decreased *NLRP3* expression in the livers of broiler chickens. The NLRs family pyrin domain containing 3 inflammasomes is a type of multiprotein innate immune complexes. Studies demonstrated that NLRP3 could regulate a variety of inflammation, pyroptosis, apoptosis, and fibrosis *via* inducing the maturation and secretion of pro-inflammatory cytokines including IL-1β and IL-6 (51, 52). Besides, NF-κB could also transcriptionally induce the activation of the NLRP3 inflammasome, resulting in cell death, pyroptosis, and apoptosis (53, 54). Consistently, our study also showed that dietary MCE supplementation decreased the *Bax*/*Bcl-2* ratio and caspase-1 levels in broiler chickens. Antiapoptotic Bcl-2 and pro-apoptotic Bax play important roles in regulating cell death (55). A high Bax/Bcl-2 ratio causes activation of caspases such as caspase-1 and caspase-3 (56). Caspase-1 is an effector caspase that can be activated in an NLRP3-dependent manner and initiate the process of pyroptosis, a type of inflammatory cell death (57, 58). A previous study showed that inhibition of hepatocyte pyroptosis could attenuate cecal ligation and puncture-induced liver injury in mice (59). Above all, our study indicated that supplementation of MCE containing protopine and allocryptopine could suppress caspase-1-induced hepatic pyroptosis through inhibiting TLR4/MyD88/NF-κB/NLRP3 inflammasome signaling pathway, which could explain the beneficial effect of MCE on liver protection in broiler chickens.

Continued active inflammation response can lead to overproduction of reactive oxygen species, causing damage to important cellular components in the aspect of lipid peroxidation, protein oxidation, and DNA histone modification (60). In this study, we found that supplementation of 0.6 mg/kg MCE or 50 mg/kg aureomycin significantly decreased MDA concentration in the liver. Free radicals are produced during normal metabolism. When the generation of free radicals exceeds the body's natural antioxidant defenses, oxidative stress will occur, causing damage to the body (61). Malondialdehyde is one of the main aldehyde products produced in the process of lipid peroxidation, and it is closely related to oxidative damage, so the degree of lipid peroxidation can be assessed by measuring the content of MDA (61, 62). Therefore, it is suggested that MCE containing protopine and allocryptopine could decrease oxidative stress of the liver of broiler chickens in this study. A previous study also found that 50 mg/kg of aureomycin decreased liver MDA concentration in broilers (63). Interestingly, relative to the CON group, dietary MCE or AGP supplementation decreased *SOD2* and *GPX1* mRNA expressions in the liver of broiler chickens. Superoxide dismutase and GPX1 are the common indicators to reflect oxidative stress (64, 65). A previous study demonstrated the addition of an inhibitor decreased antioxidant capacity in TM3 cells *via* downregulating the expressions of *SOD1* and *SOD2* through NF-κB signaling pathway (66). Meanwhile, the increase in the *SOD* and *GPX1* expression might mean the body tried to fight against emerging oxidative stress (67, 68). Therefore, the decreased *SOD2* and *GPX1* mRNA expressions in the AGP and MCE groups might be attributed to a reduction in the level of oxidative stress induced by inflammation in this study.

## Conclusion

In conclusion, dietary supplementation with 0.6 mg/kg MCE containing protopine and allocryptopine improved the growth performance and benefited liver health in broiler chickens. The beneficial effects of PFA supplementation on liver might be attributed to the inhibition of caspase-1-induced pyroptosis by inactivating the TLR4/MyD88/NF-κB/NLRP3 signaling pathway. Our study demonstrated that MCE containing protopine and allocryptopine might be an effective alternative to AGP in poultry production.

## Data Availability Statement

The original contributions presented in the study are included in the article/supplementary material, further inquiries can be directed to the corresponding authors.

## Ethics Statement

The animal study was reviewed and approved by the Care and Use Committee of Shandong Agricultural University (Ethics Approval code: SDAUA-2021-019).

## Author Contributions

YLi and HL: conceptualization and investigation. YLiu and JN: data curation and project administration. YLiu and YLi: formal analysis. NJ, LH, and SJ: methodology and software. YLiu and LY: visualization. YLiu: writing—original draft. YLi and WY: funding acquisition, validation, supervision, and writing—review and editing. All authors contributed to the article and approved the submitted version.

## Funding

This research was funded by the Major Innovative Projects of Shandong Province [Grant Number 2019JZZY020609] and Starting Research Fund from the Shandong Agricultural University [Grant Number 040/72185].

## Conflict of Interest

LY was employed by Shandong New Hope Liuhe Group Co., Ltd. The remaining authors declare that the research was conducted in the absence of any commercial or financial relationships that could be construed as a potential conflict of interest.

## Publisher's Note

All claims expressed in this article are solely those of the authors and do not necessarily represent those of their affiliated organizations, or those of the publisher, the editors and the reviewers. Any product that may be evaluated in this article, or claim that may be made by its manufacturer, is not guaranteed or endorsed by the publisher.
